# Complete genome sequence for *Slackia exigua* strain SB208, isolated from a human colonic adenocarcinoma

**DOI:** 10.1128/mra.00445-25

**Published:** 2025-09-19

**Authors:** Kaitlyn N. Lewis, Martha A. Zepeda-Rivera, Alexander A. Baryiames, Dakota S. Jones, Kaitlyn D. LaCourse, Susan Bullman, Christopher D. Johnston

**Affiliations:** 1 Genomic Medicine, MD Anderson Cancer Center 4002, Houston, Texas, USA; 2 Vaccine and Infectious Disease Division, Fred Hutchinson Cancer Center 7286, Seattle, Washington, USA; 3 Human Biology Division, Fred Hutchinson Cancer Center 7286, Seattle, Washington, USA; 4 Immunology, James P. Allison Institute, MD Anderson Cancer Center 4002, Houston, Texas, USA; University of Maryland School of Medicine, Baltimore, Maryland, USA

**Keywords:** *Slackia exigua*, colorectal cancer, PacBio, SMRT-Seq

## Abstract

We report the complete genome sequence of *Slackia exigua* SB208, isolated from the resected tumor tissue of a treatment-naïve patient with colorectal cancer. This genome is composed of a single chromosome that is approximately 2 Mb in length with an overall guanine-cytosine (GC) content of 62.5%.

## ANNOUNCEMENT

First described in the 1990s (1), *Slackia exigua*, formerly known as *Eubacterium exiguum* (2), is a gram-positive, non-spore forming, obligate anaerobic bacillus (1). Commonly isolated from the oral cavity (3–5) and associated with periodontal disease (Table 1), only 11 unique *Slackia exigua* isolate genomes and metagenomes were previously available at the National Center for Biotechnology Information (NCBI). Here we report the complete genome assembly for *Slackia exigua* SB208 isolated from a colorectal cancer (CRC) tumor tissue from a treatment-naïve patient.

**TABLE 1:** Names and associated metadata for publicly available *Slackia exigua* genome assemblies used for ANI and phylogenetic analyses^*b*^

Strain ID	GenBank accession no.	Isolation source	Assembly level
SB208	GCA_049191205.1	Colorectal tumor	Complete genome
ATCC 700122 ^*a*^CIP105133T and NCTC12994	GCA_000162875.1	Necrotic pulp samples of human deciduous teeth	Scaffold
UMB0191	GCA_030223695.1	Catheter urine	Contig
EYE_7	GCA_023148135.1	Ocular surface of a healthy person	Scaffold
DFI.6.114	GCA_024462625.1	Fecal sample	Contig
ERR589663_bin.38_CONCOCT_v1.1_MAG	GCA_938037195.1	Human gut metagenome	Contig (MAG)
SRR19171293_bin.1_MetaWRAP_v1.3_MAG	GCA_963541115.1	Oral metagenome	Contig (MAG)
SRR23388435_bin.23_MetaWRAP_v1.3_MAG	GCA_963550055.1	Oral metagenome (subgingival plaque)	Contig (MAG)
ASM4842916v1	GCA_048429165.1	Human gut metagenome	Contig (MAG)
DRR247342_bin.38_MetaWRAP_v1.3_MAG	GCA_963535765.1	Oral metagenome	Contig (MAG)
SRR10258546_bin.6_metaWRAP_v1.3_MAG	GCA_947086745.1	Vaginal metagenome	Contig (MAG)
MAG2438	GCA_048190685.1	Human gut metagenome	Contig (MAG)

^*a*^ Strain ID CIP105133T (GCF_965136305.1) and NCTC12994 (GCF_900450505.1) refer to the same type strain, ATCC 700122, in the NCBI database under different identifiers. They were excluded from ANI and phylogenetic analyses to avoid redundancy.

^*b*^ ATCC, American Type Culture Collection.

**Fig 1:**
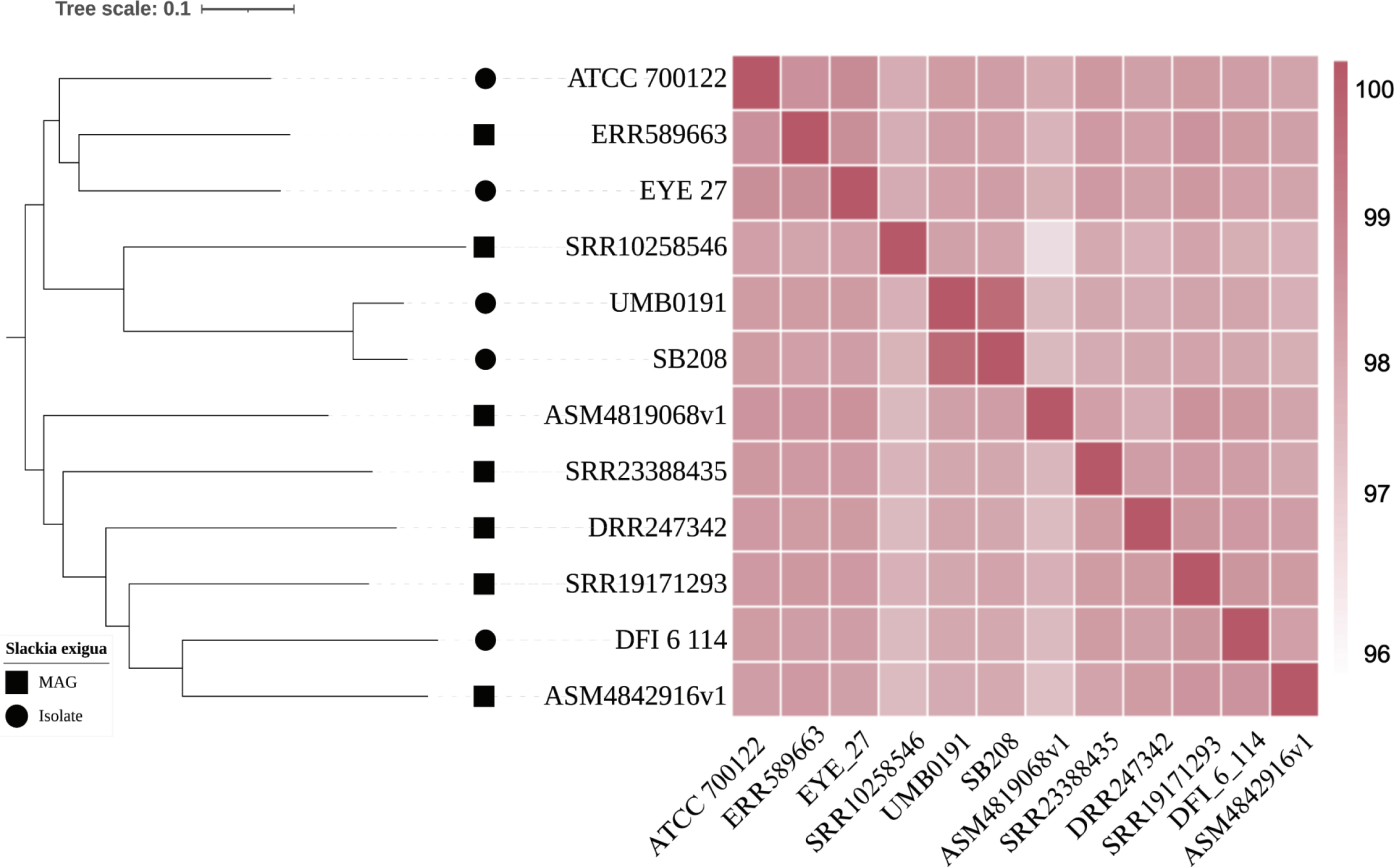
Average nucleotide identity (ANI) between *Slackia exigua* genomes with a corresponding reference-free whole-genome phylogenetic tree. The figure displays (left) a kSNP4 (10) whole-genome phylogenetic tree (kmer = 17, FCK = 0.485) of *Slackia exigua* genomes (*n* = 12), with (right) corresponding FastANI comparison values between pairs of genomes. Tree end points indicate the genome assembly level (square, MAG; circle, isolate). ANI heatmap was generated with pheatmap R package, RStudio (v.2024.12.0+467). The tree was visualized using iTOL (11), and the figure was made with BioRender.

Resected tumor specimen was plated on fastidious anaerobic agar (Oxoid, Thermo Fisher Scientific) and supplemented with 10% defibrinated horse blood (Lampire Biological Laboratories, Fisher Scientific) in anaerobic conditions at 37°C (AnaeroGen Gas Generating Systems, Oxoid, Thermo Fisher Scientific) for 72 hours. For initial identification, the 16S rRNA gene of SB208 was PCR-amplified for 35 cycles with an annealing temperature of 50°C and an extension time of 88 seconds using the 27F and 1492R primers. The sequencing results were analyzed by BLAST, indicating a top hit to *Slackia exigua* ATCC 700122 (NR_024952.1) with 96.72% similarity. Grown under the above conditions, SB208 high-molecular-weight DNA was extracted utilizing the MasterPure DNA Purification Kit (LGC Biosearch Technologies, Epicenter, Lucigen). DNA was sheared as previously described (6) and size-selected for fragments of >5 kb using BluePippin (Sage Science). Single-molecule real-time sequencing (7) with base kinetics was performed on a PacBio Sequel I instrument (Pacific Biosciences). Sequencing reads were quality filtered and processed using Microbial Assembler in the Pacific Biosciences’ SMRTAnalysis pipeline (v.9.0.0.92188) with default parameters. This generated 503,541 subreads, of which 475,900 passed quality control and were included in genome assembly, which showed an *N*_50_ value of 321.9 kb nucleotides with a mean coverage of 1,938×. This resulted in a single, circular chromosomal contig with a size of 1,987,822 nucleotides and a guanine-cytosine (GC) content of 62.5%. Analysis of the final SB208 assembly against the Genome Taxonomy Database (GTDB) using GTDB-tk (8, 9) (v.2.3.2) confirmed classification as *Slackia exigua*. Average nucleotide identity (ANI) and phylogenetic analysis (10) against publicly available *Slackia exigua* genomes indicated a close similarity to strain UMB0191 with comparative ANI values of 99.3782 and 99.3972 (Fig. 1; Table 1).

Analysis of SB208 via the NCBI Prokaryotic Genome Annotation Pipeline (PGAP) (12) (v.6.1) under default parameters annotated 1,758 genes, 1,699 coding sequences, and 59 RNAs. Both PGAP and PADLOC (13) identified a CRISPR-Cas system. PADLOC further predicted a type II restriction-modification system. In agreement with this, REBASE (14) analysis of SB208 methylome data correspondingly identified a single AGTA^m4^CT-modified nucleotide motif. Putative antibiotic resistance genes were identified using the Resistance Gene Identifier (15) (v.6.0.5) against the Comprehensive Antibiotic Resistance Database (15) (v.4.0.1). Strict hits showed that SB208 harbors putative *nimI* (5-nitroimidazole) and *vanT* (glycopeptide) resistance genes.

With limited *Slackia exigua* genome assemblies publicly available, it is notable that SB208 is a complete assembled genome isolated from a cancer-associated niche (Table 1). Therefore, the use of this isolate and its genome in future mechanistic studies may help advance our clinical understanding of this organism.

## Data Availability

The SB208 amplified 16S rRNA gene sequence (PX105876) is deposited in GenBank. SB208 raw sequencing reads (SRR33570754) and assembled genome (CP096601.1) were deposited at the National Center for Biotechnology Information under BioSample number SAMN27745308. Base modification files were submitted with the GenBank accession and methylome analysis is available at REBASE under organism number 90767. The BioProject accession number for this genome is PRJNA549513.
